# Improving Circularity via Chemical Recycling to all Rings

**DOI:** 10.1002/anie.202502436

**Published:** 2025-03-22

**Authors:** Vincent Nieboer, Karin Odelius, Peter Olsén

**Affiliations:** ^1^ Department of Fibre and Polymer Technology KTH Royal Institute of Technology Stockholm Sweden; ^2^ Wallenberg Wood Science Center Stockholm Sweden; ^3^ Laboratory of Organic Electronics Department of Science and Technology Linköping University Norrköping Sweden

**Keywords:** Equilibrium, Recyclable polyester, Recycling, Ring‐opening polymerization, Thermodynamics

## Abstract

Aliphatic polyesters synthesized via ring‐opening polymerization (ROP) have properties competitive to incumbent plastic (PE, PP), while simultaneously opening up for chemical recycling to monomer (CRM). However, not all aliphatic polyesters are prone to undergo CRM, and the ability to shift the equilibrium between polymer and monomer is tightly associated with the initial monomer structure. The standard strategy to measure CRM is to evaluate the change in free energy during polymerization (∆G_ROP_). However, ∆G_ROP_ is only one‐dimensional by assessing the equilibrium between initial monomer and polymer. But under active catalytic conditions, the depolymerization of polymers can lead to formation of larger rings, such as dimers, trimers, tetramers, and so on, via the ring‐chain equilibrium (RCE), meaning that the real thermodynamic recycling landscape is multi‐dimensional. This work introduces a multi‐dimensional chemical recycling to all rings (CRR) via a highly active catalytic system to reach RCE. Thermodynamically ∆G_RCE_ is completely different from ∆G_ROP_. Using ∆G_RCE_ instead of ∆G_ROP_ allows us to achieve CRR for polymers notoriously difficult to achieve CRM for, as exemplified within by CRR for poly(ε‐caprolactone), poly(pentadecalactone), and mixed polymer systems. Overall, this work provides a new general concept of closing the material loop.

## Graphical abstract

The initial monomer free energy of polymerization (∆G_ROP_) is traditionally regarded as the benchmark for the polymer's chemical recyclability. But if we instead consider every ring size present at equilibrium (∆G_RCE_), a different viewpoint emerges. We found that polymers previously regarded as unsuitable for chemical recycling are highly chemically recyclable at low temperatures under the right concentration and catalytic conditions.

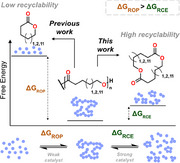

## Introduction

Recycling techniques revitalizing plastic waste are essential for a more circular material economy. Mechanical recycling is the most utilized plastic recycling approach; however, less than 9% of plastics is returned to the market as secondary plastics in the European Union.^[^ ^1^, ^2^ ^]^ The substantial effort required to clean and separate waste plastics hinders progress, but most critically, second cycle plastics are downgraded in terms of properties.^[^ ^3^, ^4^ ^]^ Specifically, mechanical reprocessing leads to side reactions via oxidation, chain scission, and cross‐linking, drastically changing the material properties.^[^ ^5^, ^6^, ^7^ ^]^ To complement mechanical recycling, chemical recycling to monomer (CRM),^[^ ^8^, ^9^, ^10^, ^11^, ^12^, ^13^, ^14^, ^15^, ^16^, ^17^, ^18^ ^]^ could, provide an alternative pathway for infinite recycling cycles and countless generations of plastic as the properties would be indistinguishable from the virgin material. In this field, monomers polymerized via ring‐opening polymerization (ROP) are highlighted as particularly promising.^[^ ^19^, ^20^ ^]^

The success of CRM relates to the thermodynamics of the polymerization‐depolymerization equilibrium. ROP is an equilibrium reaction between the monomer and polymer, where the initial monomer structure, concentration, reaction temperature along with solvent type dictate the equilibrium monomer concentration ([M]_eq_). Much recent activity comprising five‐membered lactones,^[^ ^15^, ^21^, ^22^ ^]^ monomers with triggered recyclability,^[^ ^23^, ^24^ ^]^ thiolactones,^[^ ^25^, ^26^ ^]^ and other sophisticatedly designed structures have been discussed as they are well suited for CRM. The thermodynamics of ROP is described as the change in entropy (∆S) and enthalpy (∆H) during polymerization, with the ceiling temperature (T_c_, at ∆G = 0) defined as T_c_ = ∆H/∆S, has become the standard way to assess the CRM of polymers.^[^ ^11^, ^17^, ^25^, ^27^, ^28^, ^29^, ^30^, ^31^, ^32^, ^33^, ^34^ ^]^ The unfortunate consequence of a low T_c_ and thereby polymers considered prone to undergo CRM are their sensitivity to changes in their surrounding environment. This correlation is disadvantageous as, on the one hand, we want high CRM for circularity, and on the other hand, we want low CRM for predictable and stable material properties.

Catalysts do not change the system's thermodynamics but rather the rate at which it reaches equilibrium. However, if the catalytic system it too weak the time frame to reach all equilibrium in the system will to very long. Anionic initiation of ROP leads to the formation of rings larger than the monomer for both polyesters and polycarbonates in equilibrium,^[^ ^35^, ^36^, ^37^, ^38^, ^39^, ^40^, ^41^, ^42^ ^]^ in accordance with the ring‐chain equilibrium (RCE). In our recent work on anionic polymerization of unstrained and strained lactones, we also found larger rings of varying sizes in equilibrium.^[^ ^43^ ^]^ These results reveal the importance of a highly active catalytic systems to reach all equilibriums in the system, and the question is how does ΔG_ROP_ (equilibrium between initial monomer and polymer) and ΔG_RCE_ (equilibrium between all rings and polymer) translate into difference in chemical recyclability. Perhaps we have not revealed the full potential of chemical recycling of polymers by considering only the thermodynamics of ROP and not of the full thermodynamics of RCE. To exemplify, ε‐caprolactone (εCL) has a T_c_ of 1787°C,^[^ ^44^ ^]^ meaning it is less suitable for CRM as there is very little initial monomer in equilibrium at any practical temperature.^[^ ^38^, ^45^ ^]^ However, from the perspective of RCE, PεCL converts almost entirely into rings consisting of multiple repeating units at low concentrations.^[^ ^38^, ^39^ ^]^ This underlines the difference since, ΔG_ROP_ is one‐dimensional, only taking the initial monomer and polymer equilibrium into consideration. In contrast, ΔG_RCE_ is multi‐dimensional, looking at the equilibria of all rings with the polymer.

**Figure 1:**
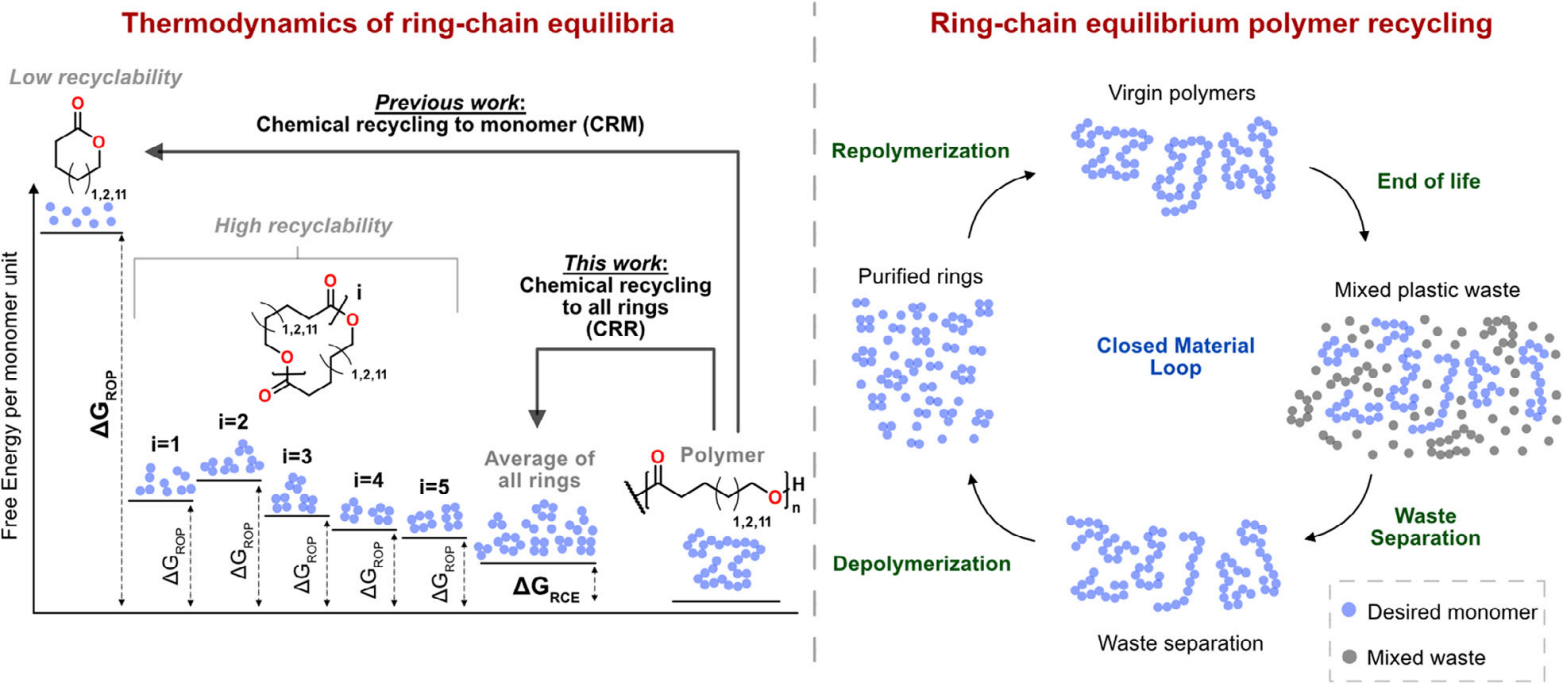
Schematic representation of a) the thermodynamics of the entire ring‐chain equilibria and b) polymer recycling through the entire ring‐chain equilibria.

In this work we hypothesize that a highly active catalyst, such as *^t^* BuOK used for anionic ROP, would enable us to reach ΔG_RCE_ equilibrium within practical time frames (Figure 1). If this holds, it will provide an alternative strategy to chemically recycle polymers, not via CRM, but instead chemical recycling to all rings (CRR). Our aim is using the full thermodynamics of the system via the RCE to enable chemical recycling to all rings instead of the initial monomer to polymer equilibrium that traditional ROP thermodynamics provide. The study is centered around three monomers with vastly different polymerization behavior and CRM via ΔG_ROP_, namely εCL, δ‐valerolactone (δVL), and pentadecanolide (PDL). We put great emphasis on constructing the entire thermodynamic landscape of all the rings in equilibrium via ΔG_RCE_ and to assess their independent contribution to the overall CRR of the polymer.

## Results and Discussion

Ring‐opening polymerization (ROP) of an initial monomer and the full ring‐chain equilibrium (RCE) covers very different breaths of the thermodynamic landscape. Each ring follows an independent process with unique thermodynamics and energy barriers. Thus, our ability to fully reach the RCE depends on the catalytic system used during polymerization. Plenty of values for ROP thermodynamics exist and are regarded as the measure of the system's chemical recyclability,^[^ ^11^, ^17^, ^23^, ^25^, ^27^, ^28^, ^29^, ^30^, ^31^, ^32^, ^33^, ^34^ ^]^ but, these values are one‐dimensional, looking only at the equilibrium between polymer and the initial monomer structure, and thus does not truly reflect the full chemical recyclability potential of the systems if we were to consider chemical recycling to all rings (CRR).

To assess the generality of CRR, three systems based on three different monomers with different polymerization behavior were selected; δVL: high polymerization rate and high [M]_eq_, εCL: high polymerization rate and low [M]_eq_ and PDL: low polymerization rate and low [M]_eq_. First, we study and compare the thermodynamics of RCE to ROP and then proceed to chemically recycle all polymers via CRR, and finally close the loop by reforming PεCL.

### Expanding From ROP to RCE Thermodynamics Through Catalyst Choice

**Figure 2:**
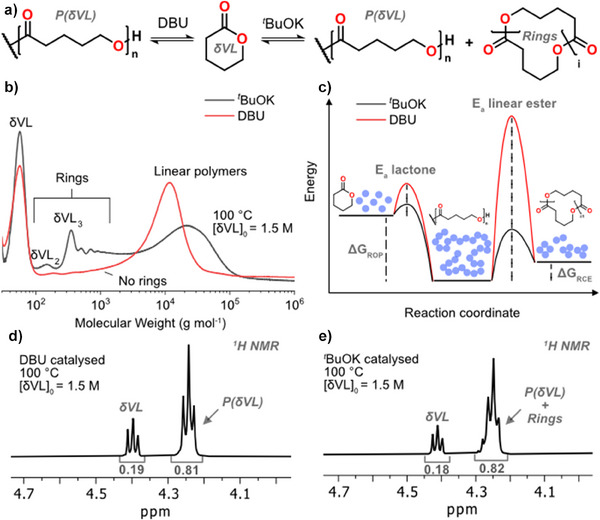
a) Reaction scheme outline equilibria obtained with either DBU or ^t^BuOK. b) SEC traces of the equilibrium obtained in the polymerization of [VL]_0 _= 1.5 M at 100°C in toluene using ^t^BuOK or DBU. c) Schematic representation of difference in activation energy. ^1^H NMR spectra for δVL polymerized to equilibrium using either d) DBU or e) ^t^BuOK.

The catalyst does not change the thermodynamics of the system. However, each ring to polymer equilibrium is associated with its independent energy barrier, meaning the kinetics towards equilibrium depend on the catalyst. To emphasize the difference in the equilibrium processes for ROP and RCE, we polymerized δVL at 100°C with two different catalytic systems of different activity, 1,8‐diazabicyclo[5.4.0]undec‐7‐ene (DBU) base with BnOH as initiator and potassium tert‐butoxide (^t^BuOK). For the less active DBU‐BnOH system, δVL monomer and PδVL polymer were solely detected, while for the more active ^t^BuOK rings of various δVL_n_ sizes larger than the initial δVL monomer was generated, revealing that the RCE was established (Figure 2a,b). In our recent findings we found that *^t^* BuOK polymerizes nearly strainless macrolactones, such as PDL, exceptionally fast,^[^ ^43^ ^]^ compared to DBU that is unable to yield any conversion of PDL.^[^ ^46^ ^]^ The interpretation is that *^t^* BuOK enables activation energies sufficiently low for unstrained monomers or linear esters to react, meaning it also enables the reverse reaction to form rings larger than the initial monomer towards the RCE (Figure 2c). In both systems, similar equilibrium monomer concentration [VL]_eq_ was found regardless of catalytic system as determined by ^1^H NMR spectroscopy (Figure 2d–e and Figure S1), underlining that the RCE thermodynamics with all rings is an extension of traditional ROP thermodynamics and each ring needs to be regarded as an independent ring‐chain equilibrium. We found that *^t^* BuOK was highly efficient to establish the RCE in less than 3 h for all polymer systems; PδVL, PεCL, and PPDL (Figure S2). Considering the broader perspective, these findings are not only related to these specific monomer/polymer structures, as a similar strategy was recently adopted for forming chemically recyclable ketal‐esters through in‐situ ring formation.^[^ ^23^ ^]^

An analytic challenge is that many of the larger rings formed by the RCE overlap with the polymer peak in the ^1^H NMR spectra. Hence, a more straightforward method to assess macrocycle content is from SEC traces. RCE determination by SEC has been shown to agree well with the actual concentration of rings in equilibrium,^[^ ^38^, ^47^ ^]^ and we found similar values when comparing the equilibrium concentration of the monomeric lactone determined from ^1^H NMR spectroscopy versus those determined by SEC (Figure S4).

### Modulating the Equilibrium of RCE for Chemical Recycling to all Rings

**Figure 3:**
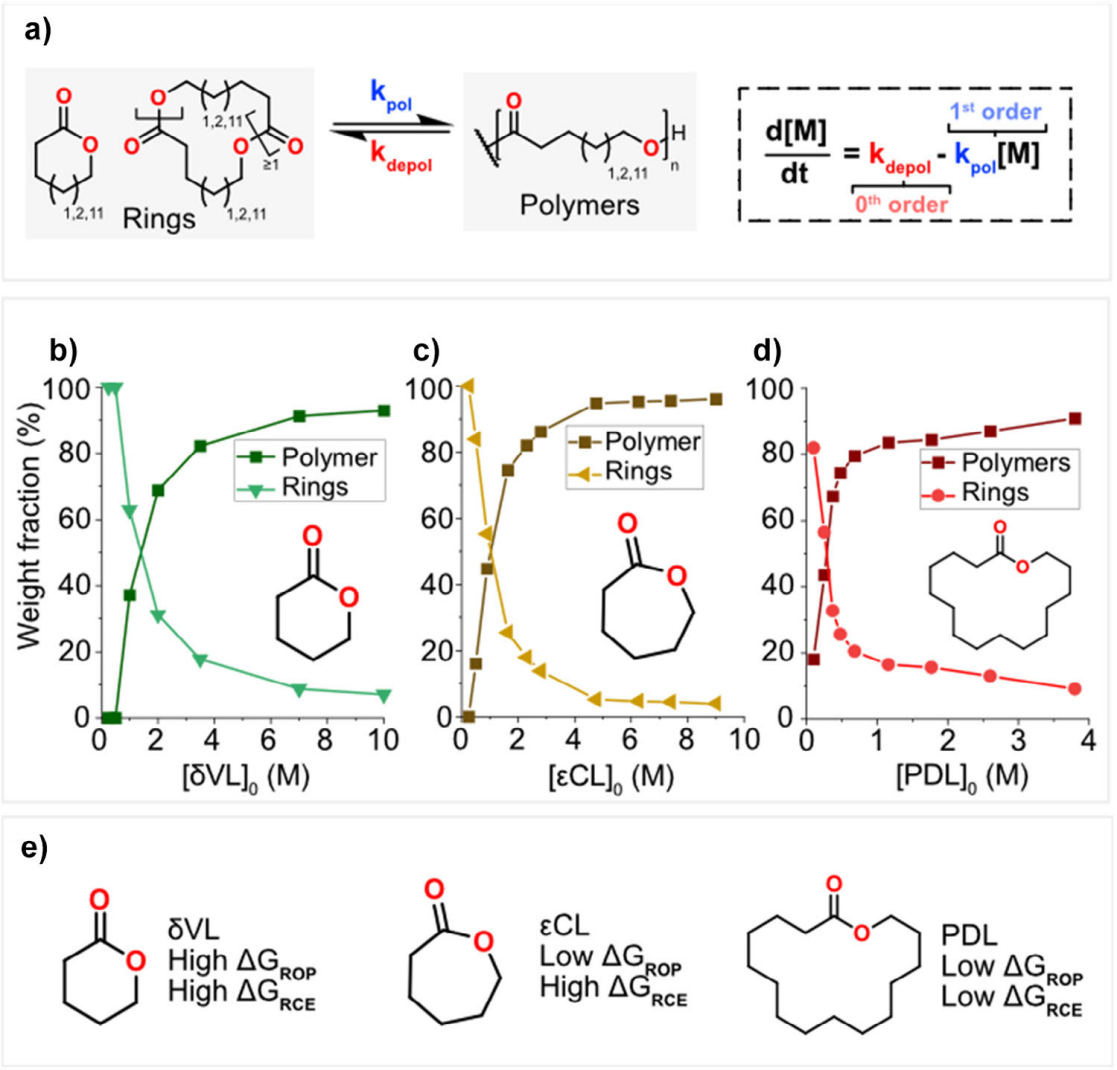
a) Schematic representation of ring‐chain equilibria with associated kinetics. Weight fraction of all rings and polymer chains at different starting concentrations of b) δVL, c) εCL, and d) PDL. e) summary of ∆G_ROP_ and ∆G_RCE_ for each monomer.

The adopted strategy to regulate ring‐polymer concentrations in CRR is to change the system's free energy by initial monomer concentration or temperature. Transesterification with anionic initiators, such as *^t^* BuOK, mainly occurs through an intramolecular transesterification (back‐biting) pathway, where the interpolymer transesterification route is suppressed.^[^ ^43^ ^]^ To study the influence of dilution on RCE, that is, the formation of all ring‐sizes; δVL, εCL, or PDL were polymerized at different initial monomer concentrations in toluene with *^t^* BuOK at 100°C. Once equilibrium was reached, we analyzed the SEC elugrams (Figure S5). Changing the initial monomer concentration significantly altered the weight fraction of the linear polymers‐to‐rings formed for all monomer structures (Figure 3b–d). It is important to note that depolymerization follow (pseudo) zero‐order kinetics whereas polymerization follows first‐order dependent kinetics (Figure 3a), however, at the same temperature, the ratio between the rings are constant (Figure S5). At 0.25 M the PεCL sample consists entirely of rings and no linear polymers, irrespective of temperature (Figure 3c and S6A). Contrary, at 0.25 M PPDL renders a ring weight fraction of about 60% with a significant portion of linear polymers (Figure 3d and S6B). Continuing to dilute the PPDL system to 0.1 M increases the weight fraction to 80%, but it does still not reach 100%. These results show that each ring‐size and chemical structure is associated with specific formation kinetics and thermodynamic stability;, that is, PPDL has lower kinetics and higher thermodynamic stability than PεCL. These observations correlate to the Jacobson‐Stockmayer theory, which predicts that larger rings are less prone to form scaling with n^−2.5^,^[^ ^48^ ^]^ where n is the number of atoms in the ring. The smallest ring that can be formed with low ring‐strain for PPDL is its monomer (*n* = 16 atoms) followed by the dimer (*n* = 32 atoms), whilst εCL can form dimers (εCL_2_ *n* = 14 atoms), trimers (εCL_3_ *n* = 21 atoms), and tetramers (εCL_4_ *n* = 28 atoms) from the same number of atoms in the ring. Hence, the rings with fewer atoms are statistically more likely to form. This concept is easily observed when the total weight fraction of rings for the three studied monomers are overlaid, as δVL and εCL show much higher weight fractions of rings than PDL at the same concentrations (Figure S7A). As depolymerization is (pseudo) zero‐order, adding more monomer than the equilibrium ring weight fraction leads to polymerization, that is, the incorporation of all excess monomer into the linear polymer and hence an increased chain length is observed with increased starting monomer concentration (Figure S7B). From the equilibrium ring weight fraction, we can establish that δVL and εCL show similar potential for chemical recycling in RCE, despite having vastly different CRM (Figure 3e).

### The Thermodynamic Landscape of all Rings

**Figure 4:**
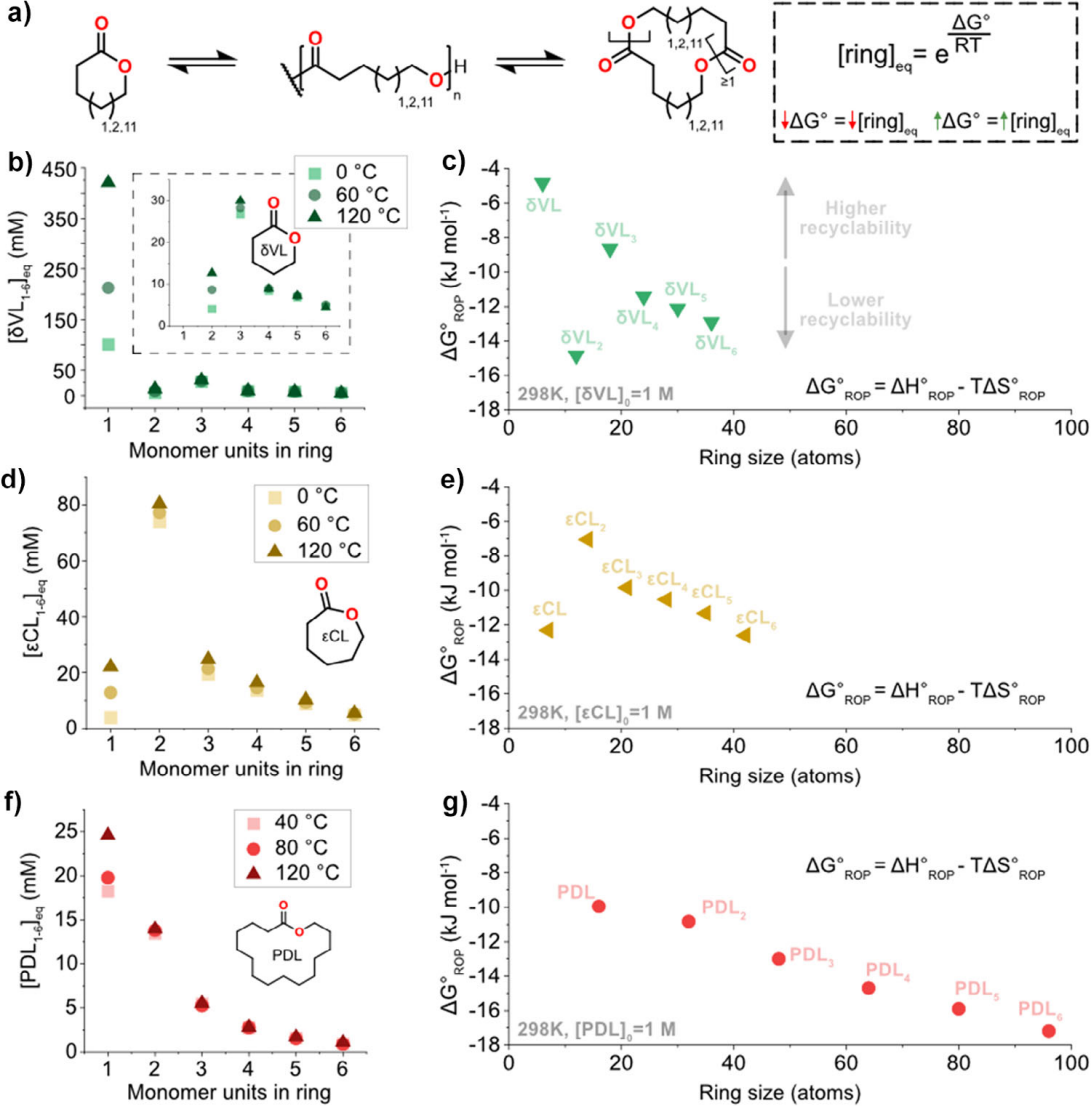
a) schematic representation of the ring‐chain equilibria. Equilibrium concentrations versus rings at different temperatures for b) δVL, d) εCL, and f) PDL. Free energy of ring‐opening at 298 K (25°C) and 1 M starting monomer concentration for c) δVL, e) εCL, and g) PDL.

To assess the chemical recycling to all rings via RCE, we decided to map the individual thermodynamic features of each ring and globally assess the overall thermodynamics of the system (ΔG_RCE_). The mass balance between rings and linear polymers is highly dependent on the concentration; however, the ratio between the different rings is constant at the same temperature regardless of the starting monomer concentration. These results show that each ring is associated with a specific energy barrier. Hence, changing the temperature at a particular concentration enables us to map the thermodynamic equilibrium of the rings of different sizes for the three different systems (δVL, εCL, and PDL). We determined the concentrations of all mono and multimeric rings up to the hexamer with varying temperatures and used van ‘t Hoff plots to gain insights into the ∆H_ROP_° and ∆S_ROP_° parameters for each ring and reveal which ring that contributes most in changing the free energy of the system, hence drive polymerization or depolymerization (Figure 4b,d,f, and Figure S8). The data revealed a complex thermodynamic landscape, where some rings are strained, that is, δVL, δVL_2_, PDL, εCL, and εCL_3_, whereas most rings are strainless. We find a correlation close to the theoretical i^−2.5^, predicted by the Jacobson‐Stockmayer theory,^[^ ^48^ ^]^ between the concentration of each ring and the ring size (i = 2, 3, … 6, i.e., dimer, trimer, … hexamer) with only a slight deviation derived from non‐theta experimental conditions (Figure S9). Thus, larger rings are strainless and obey Gaussian conformation statistics, which agrees with previous work on macro‐ring equilibrium formation for εCL and δVL.^[^ ^38^, ^39^, ^47^ ^]^ Moreover, the calculated ∆S_ROP_° values are in excellent agreement with experimental work on lactones of different ring sizes including δVL, εCL, and PDL by Lebedev^[^ ^49^ ^]^ and Ito and co‐workers^[^ ^38^, ^39^ ^]^ (Figure S10). With the obtained ∆H_ROP_° and ∆S_ROP_° values, we can model the free energy of ring‐opening for each ring (Figure 4c,e,g). The closer ∆G_ROP_ is to 0 kJ mol^−1^, the higher the equilibrium concentration of that ring and, thus, the higher the recyclability. ∆G_ROP_ shows that the rings δVL and εCL_2_ contribute immensely towards chemical recycling due to their low ∆G_ROP_ in their respective polymer system. The consensus in the field is that PδVL is more chemically recyclable to monomer than PεCL. However, this might not hold when we perform chemical recycling to all monomers via RCE and consider the entire thermodynamic landscape.

**Figure 5:**
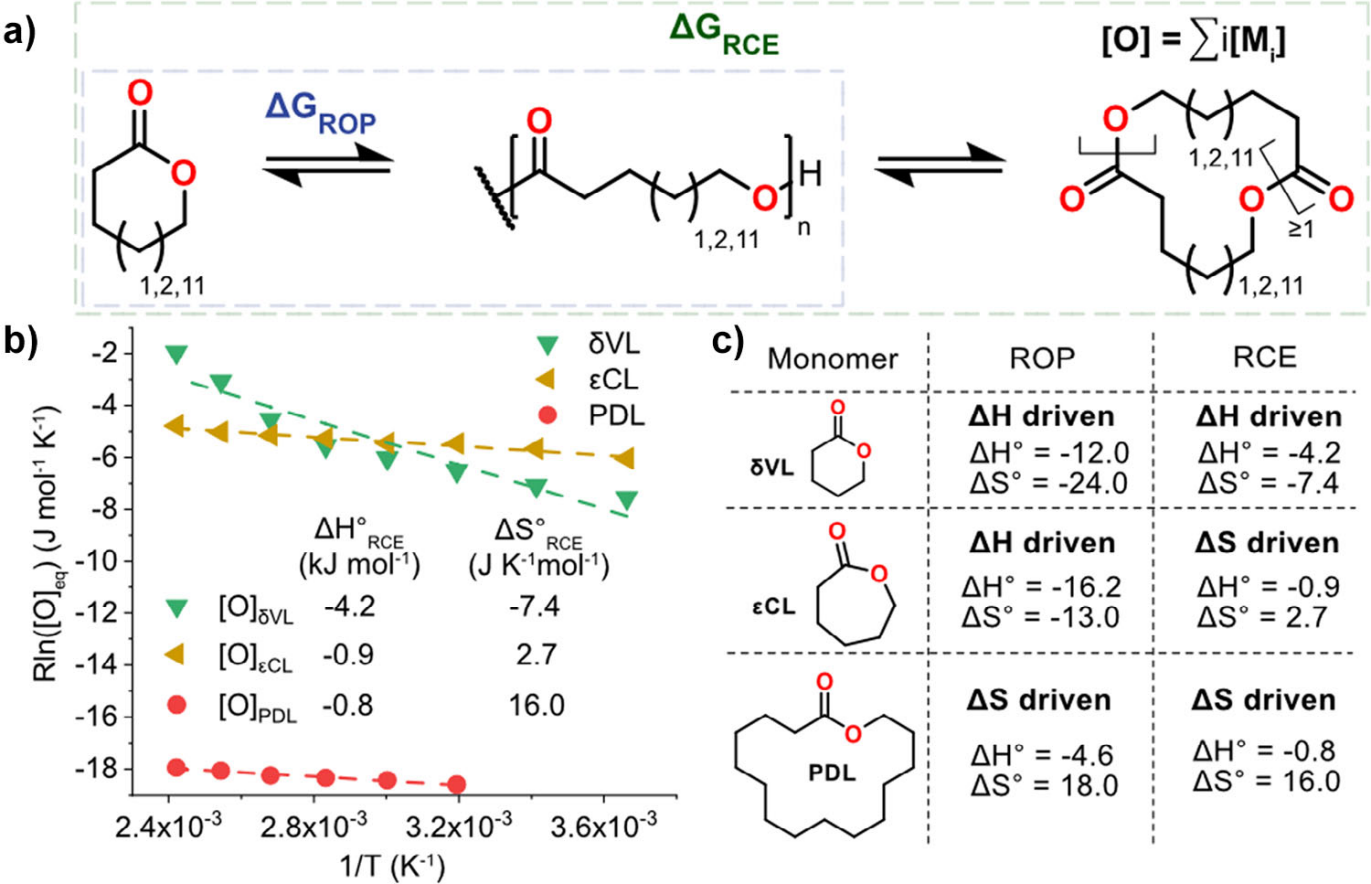
[O]_eq_ is the weight fraction of rings times the starting monomer concentration and i is the number of monomer units per ring. a) Mechanism for monomer and larger ring formation. b) Equilibrium concentration of all rings versus temperature for the determination of average polymerization thermodynamic parameters of all rings. c) Summary of driving force of ring‐opening and polymerization for δVL, εCL, and PDL, ΔH^0^ is in kJmol^−1^ and ΔS^0^ is in JK^−1^ mol^−1^.

To gain a better insight into the total chemical recyclability to all rings (CRR) via the RCE of our studied systems, we analyzed the equilibrium weight fraction of all rings and multiplied it by the total initial monomer concentration, denoting the result [O]_eq_ (Figure 5a), at different temperatures (Figure 5b). Enthalpy plays a small role in the RCE, sharply contrasting the parameters for ROP of the initial monomer units δVL and εCL (Figure 5c). Thus, looking at the all rings thermodynamics ΔG_RCE_ of the system, it is clear that a new view of the thermodynamics emerges. For PδVL, with the initial monomer δVL having a high equilibrium concentration, the ΔG_RCE_ is enthalpy driven; however, in the case of PεCL, the initial monomer εCL only represents a small fraction of all rings in equilibrium (Figure S11), making the overall ΔG_RCE_ entropy‐driven. The consequence is that PεCL is more chemically recyclable at low temperatures compared to PδVL (Figure 5b and Figure S12). Specifically, PεCL will be chemically recyclable to a greater extent at temperatures < 330 K (57°C), derived from the cross‐over of [O]_eq_ (Figure S12), whereas PδVL is depolymerized to a greater extent at temperatures > 330 K (57°C). These results highlight the power of chemical recycling through the RCE of polymers consisting of monomers with high ceiling temperatures. In the case of PPDL, both initial ΔG_ROP_ and ΔG_RCE_ remained entropy‐driven and [O]_eq_ is comparably lowest at all analyzed temperatures.

### Chemical Recycling to all Rings via the RCE

The complete chemical recycling to all rings (CRR) via the RCE was studied for all polymer systems, PδVL (117.8 kg mol^−1^, Ð = 2.04), PεCL (129.0 kg mol^−1^, Ð = 2.10), and PPDL (102.5 kg, mol^−1^, Ð = 1.75) at 0.25 M in toluene using 1 mol% of *^t^* BuOK. The rings were purified from residual linear oligomers by passing through a small silica pad (Figure S13) and then concentrated in vacuo to remove the toluene. SEC and ESI‐MS characterization indicated that the samples consisted entirely of rings (Figure S14). In every case, about ∼ 95% of the toluene could be recovered through distillation. Respectively, 96%, 93%, and 41% by weight of the rings, consisting of the monomeric and larger cyclic esters, could be isolated for PδVL, PεCL, and PPDL. The seemingly low yield for PDL_n_ rings relates to the RCE thermodynamics of PPDL depolymerization at that concentration. An important aspect of CRR via RCE is that it is also applicable to mixed polymer systems. To exemplify, a mixture of PPDL and PδVL was depolymerized under the same conditions as the homopolymers, with an overall yield of 78% of rings of various sizes including rings containing both monomers, as confirmed by ESI‐MS (Figure S15 and Table S1). Comparably, simultaneous depolymerization reveals a synergistic effect in terms of chemical recyclability, where the mixed system of PPDL‐PδVL outperforms the homopolymer systems in terms of total yield. These results may be attributed to the overall change in RCE thermodynamics of the simultaneous depolymerization system, compared to the homo(de)polymerization systems (Figure S16). Excitingly, even the ROP thermodynamics of δVL were altered in the mixed system (Figure S17), which is attributed to increased competition of δVL units in the RCE, were the mixed rings had a composition of 57% δVL and 43% PDL (Table S2, Figure S18). These results cannot be derived from a classical ROP thermodynamic description of initial monomer‐polymer equilibria. Instead, RCE thermodynamics offers a unique insight to the underlying mechanism by taking all rings into account.

### Closing the Loop by Chemical Recycling to all Rings – PεCL and an Example

**Figure 6:**
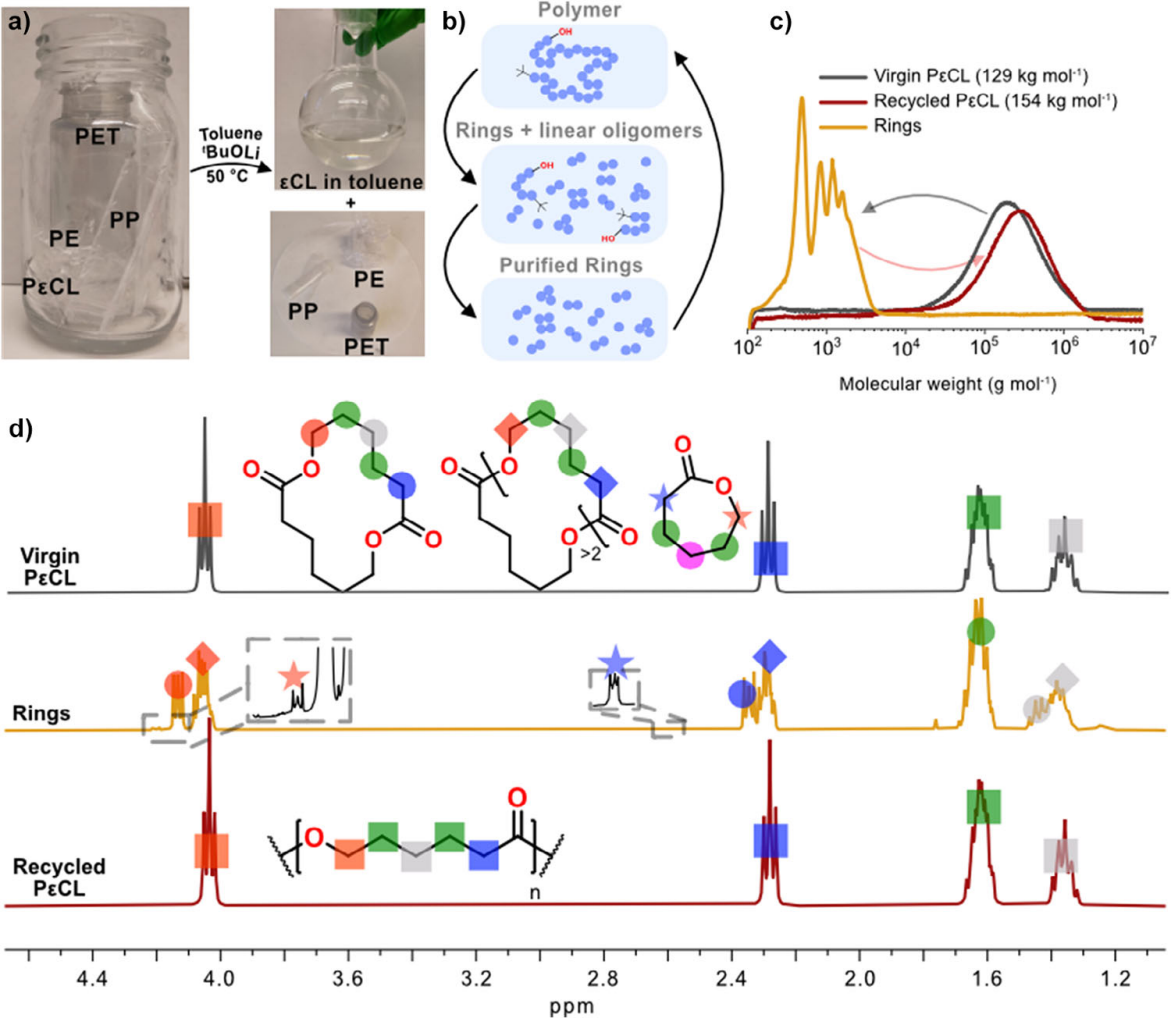
a) Depolymerization of PCL in toluene in the presence of PP, PE, and PET. b) Schematic representation of the recycling strategy. c) SEC traces of virgin PεCL (M_n_ = 129 kg mol^−1^, Ð = 2.10), purified rings, and recycled PεCL (M_n_ = 154 kg mol^−1^, Ð = 2.01). d) ^1^H NMR (CDCl_3_, 298 K) of the purified rings and PCL.

**Figure 7:**
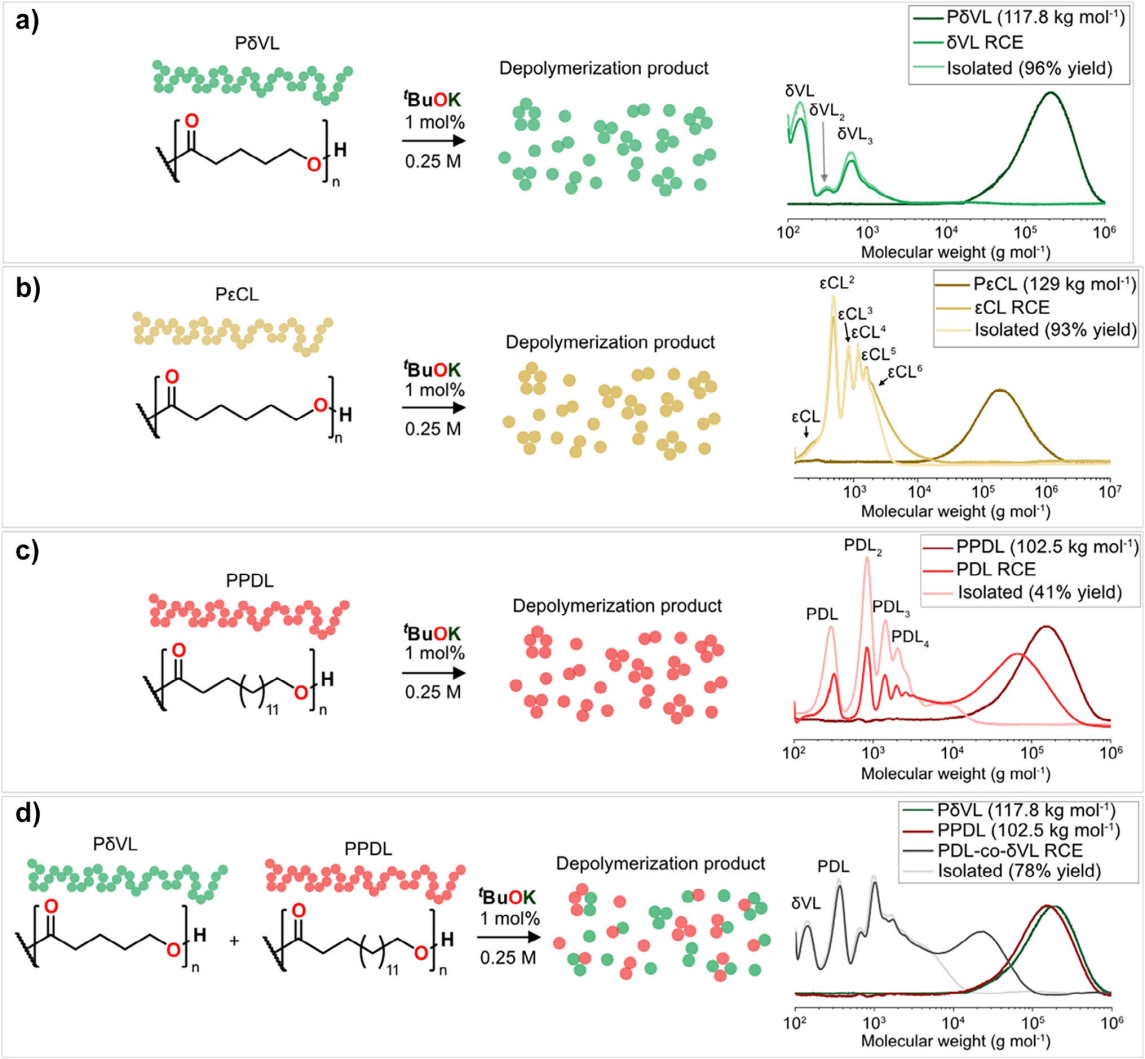
Depolymerization of polyesters in toluene with ^t^BuOK and accompanying starting polymer, RCE, and isolated ring mixture SEC traces for a) PδVL, b) PεCL, c) PPDL, and d) PδVL and PPDL polymer mixture (1:1).

To give an example of how chemical recycling to all monomers via RCE can be used from an actual cradle‐to‐cradle perspective, we extended this to a mixed plastic waste stream consisting of PET, PE, PP, and PεCL (129.0 kg mol^−1^, Ð = 2.10). We first added toluene to the plastic mixture and dissolved PεCL at 50 °C, then *^t^* BuOLi was added to depolymerize PεCL into rings at the same temperature (Figure 6a). The cyclo‐depolymerization of PεCL also yields a small fraction of linear oligomers originating from the chain ends, which were removed by passing the liquid phase over a small silica pad (Figure 6b). Concentrating the liquid phase gave a 93% yield of purified rings (Figure 6c). No characteristic alcohol end‐group signals between 3.5 and 3.8 ppm were observed by ^1^H NMR, and the monomer, dimer, and larger rings were identified (Figure 6d). The isolated mixture of εCL‐rings was repolymerized targeting a molecular weight similar to the original PεCL (recycled 154 kg mol^−1^ vs. virgin 129 kg mol^−1^) using an (CH_2_OH)_2_‐AlEt_3_ system at 120 °C for 4 h in bulk (Figure 7c). This catalytic system was selected as it is highly robust, thereby we circumvent the need of any further purification or drying of the εCL_n_‐rings to enable polymerization. The polymers were purified by precipitation in MeOH and solvent casted from dichloromethane, followed by mechanical testing. High molecular weight PδVL has been shown to have comparable tensile properties to LDPE and HDPE.^[^ ^50^ ^]^ We found the same to be true for both virgin and recycled PεCL (Figure S19; for raw tensile curves see Figure S20 and for more information on thermal properties, see Figure S21), where the respective average strain at break and Youngs modulus were 934% and 523 MPa for virgin PεCL, 937% and 537 MPa for recycled PεCL, 535% and 403 MPa for HDPE, 300% and 228 MPa for LDPE. As PE constitutes the bulk of petroleum‐based plastic; this work reveals the potential of using chemical recycling to all monomers via RCE at low temperatures (50°C) to enable a circular polymer economy without compromising properties. We believe that chemical recycling to all monomers via RCE is applicable well beyond polyesters and could include any polymer with functionalities in the main chain, such as polyolefins that contain unsaturated bonds, polyamides, polycarbonates, polyethers, and so on, granting us a new synthetic tool in our quest towards a more benign material economy.

## Conclusion

The classic chemical recycling to monomer (CRM) described by ROP thermodynamics only addresses the equilibrium between initial monomer and polymer, hence, it does not fully reflect the full potential considering chemical recycling to all rings (CRR) via ring‐chain equilibria (RCE) thermodynamics. Within, we explored the RCE thermodynamics of the monomers and subsequent polymers from δVL, εCL, and PDL and compared CRM with CRR. Under the right catalytic conditions, we can go from traditional ROP thermodynamics (∆G_ROP_) to the all ring RCE thermodynamics (∆G_RCE_). Importantly, the independent equilibrium of each ring enables us to create a complete thermodynamic landscape of all rings and their contribution to chemical recycling. Remarkably, by using ∆G_RCE_, we found that εCL contains a larger equilibrium ring content than δVL at low temperatures, suggesting that PεCL has higher recyclability potential than PδVL at low temperatures. This observation is completely counterintuitive and unexpected if only ROP thermodynamics are considered. To demonstrate the feasibility of CRR through RCE, PδVL, PεCL, PPDL, and a mixed system of PdVL and PPDL were depolymerized and the rings isolated. We also exemplified CRR for PεCL in mixed waste stream of PP, PE, and PET. Importantly, after isolation εCL_n_ could be repolymerized to high molecular weight PεCL with thermal and mechanical properties equal to the pristine material.

The concept presented within is important in the design of circular materials. We believe that the concept goes well beyond the aliphatic polyesters explored in this study. Future endeavors might include recycling multiple polymers from mixed waste streams through CRR, polymers with strategic CRR design, and other repeating unit functionality.

## Supporting Information

The authors have cited additional references within the Supporting Information.^[^ ^38^, ^49^ ^]^

## Conflict of Interests

The authors declare no conflict of interest.

## Supporting information



Supporting Information

## Data Availability

The data that support the findings of this study are available from the corresponding author upon reasonable request.
