# Supplementary material for: Improving Circularity via Chemical Recycling to all Rings
Source: Angew Chem Int Ed Engl. 2025 Mar 22;64(19):e202502436. doi: 10.1002/anie.202502436 (PMC12051835; doi:10.1002/anie.202502436)
Supplement: Supplementary file 1 — Supporting Information [file ANIE-64-e202502436-s001.docx]

# Supporting information for

**Improving Circularity via Chemical Recycling to All Rings**

Vincent Nieboer^1^, Karin Odelius^1,2^*, and Peter Olsén^2,3^*

^1^Department of Fibre and Polymer Technology, KTH Royal Institute of Technology, SE-100 44, Stockholm, Sweden.

^2^Wallenberg Wood Science Center

^3^Laboratory of Organic Electronics, Department of Science and Technology, Linköping University, Norrköping

*Corresponding authors: Karin Odelius ([hoem@kth.se](mailto:hoem@kth.se)) and Peter Olsén ([peter.olsen@liu.se](mailto:peter.olsen@liu.se))

Table of contents

[Supporting information for 1](#_Toc181366443)

[Materials 4](#_Toc181366444)

[Instrumentation 4](#_Toc181366445)

[*Size exclusion chromatography (SEC)* 4](#_Toc181366446)

[*Nuclear magnetic resonance (NMR)* 4](#_Toc181366447)

[*Tensile testing* 4](#_Toc181366448)

[*Differential scanning calorimetry (DSC)* 4](#_Toc181366449)

[*Thermal gravimetric analysis (TGA)* 5](#_Toc181366450)

[*Electron spray ionization – mass spectrometry (ESI-MS)* 5](#_Toc181366451)

[Methods 5](#_Toc181366452)

[Equilibrium studies 5](#_Toc181366453)

[Polymer synthesis 5](#_Toc181366454)

[Recycling of PεCL from mixed waste 6](#_Toc181366455)

[Data analysis 6](#_Toc181366456)

[Additional data 7](#_Toc181366457)

[Figure S1. Equilibrium δVL concentration at different temperatures in the presence of different catalysts determined using ^1^H NMR. 7](#_Toc181777222)

[*Figure S2. (A) Depolymerization of PPDL (M_n_ = 67.3 kg mol^-1^) over time with ^t^BuOK. (B) M_n_ with time for ^t^BuOK and TBD at 80 °C.* 7](#_Toc181777223)

[*Figure S3. ^1^H NMR shift of the terminal methylene CH_2_ group during the polymerization initiated with ^t^BuOK of (A) PDL and (B) εCL.* 8](#_Toc181777224)

[*Figure S4. Ratio of calculated equilibrium concentration between ^1^H NMR and SEC for δVL, εCL, and PDL monomers in toluene at different temperatures. In each case, monomer concentration was obtained from integration of the monomer signal compared to the signals of monomer+other rings+polymer.* 8](#_Toc181777225)

[Figure S5. SEC elugrams of the obtained RCE during the polymerization of εCL with ^t^BuOK at different starting concentrations. 8](#_Toc181777226)

[Figure S6. SEC traces of the equilibrium states of (a) [εCL]_0_=0.25 M, (b) [PDL]_0_=0.25, and (c) [εCL]_0_=2 M at different temperatures. 9](#_Toc181777227)

[Figure S7. (A) Overlay of ring weight fractions from Figure 3 (B)-(D). (B) M_n_ of polymer chains with increasing monomer starting concentration. 9](#_Toc181777228)

[Figure S8. Equilibrium concentration versus inverse temperature plots for determination of thermodynamic parameters for a) δVL b) εCL,, and c) PDL. 10](#_Toc181777229)

[Figure S9. Log-log plots of equilibrium concentration versus ring size (i) at 100 °C for (A) δVL, (B) PDL, and (C) εCL. 11](#_Toc181777230)

[Figure S10. The entropy of ROP for the rings of various sizes compared to literature values for unsubstituted aliphatic lactones. Literature data can be found in Ref. 1 and 2. 11](#_Toc181777231)

[Figure S11. Fraction of monomer out of all rings at equilibrium at different temperatures. Predicted lines were derived from thermodynamics (∆H and ∆S). 11](#_Toc181777232)

[Figure S12. Concentration of monomer present in rings ([O]) at equilibrium for different temperatures. Predicted lines were derived from thermodynamics (∆H and ∆S). 12](#_Toc181777233)

[Figure S13. Filtration of reaction crude containing rings and short linear oligomers to remove linear oligomers. 12](#_Toc181777234)

[Figure S14. ESI-MS mass spectra of isolated (A) δVL rings, (B) PεCL rings, and (C) PDL rings. 13](#_Toc181777235)

[Figure S15. ESI-MS mass spectra of isolated rings obtained from PPDL-PδVL co-depolymerization. 14](#_Toc181777236)

[Figure S16. van ‘t Hoff plot for the determination of the enthalpy and entropy of the RCE during co-depolymerization of PδVL and PPDL. 15](#_Toc181777237)

[Figure S17. Van ‘t Hoff thermodynamic plot for the determination of δVL ROP parameters during homo polymerization and co-polymerization with PDL systems. Reactions were performed in toluene with 1 mol% ^t^BuOK as initiator. 15](#_Toc181777238)

[Figure S18. ^13^C NMR spectra (101 MHz, CDCl_3_) of isolated δVL-PDL rings during simultaneous depolymerization of a mixture of PδVL and PPDL in the presence of ^t^BuOK in toluene. 17](#_Toc181777239)

[Figure S19. Stress-strain curves of virgin PεCL, recycled PεCL, HDPE (MFI = 8), and LDPE (MFI = 5) from tensile testing (6 mm/min, 298 K). 17](#_Toc181777240)

[Figure S20.Raw tensile curves (6 mm/min, 298 K) for (A) HDPE, (B) LDPE, (C) recycled PεCL, and (D) virgin PεCL. 18](#_Toc181777241)

[Figure 21. Thermal analysis of PεCL, recycled PεCL, LDPE, and HDPE using (A) TGA (N_2_, 10 K min^-1^) and (B) DSC (N_2_, 10 K min^-1^). 18](#_Toc181777242)

## Materials

*^t^*BuOK 1 M in THF, *^t^*BuOLi 1 M in THF, anhydrous toluene, ethylene glycol, and AlEt_3_ 1 M in hexane were all obtained from Sigma Aldrich and stored inside a nitrogen filled glovebox. HPLC grade chloroform stabilized with amylene (Sigma Aldrich) and CDCl_3_ (Sigma Aldrich) was stored under ambient conditions. Monomers δ-valerolactone (δVL) (Sigma Aldrich, technical grade), pentadecanolide (PDL) (98% Sigma Aldrich), and ε-caprolactone (εCL) (Sigma Aldrich, 99%) were all dried over P_2_O_5_ for 18 hours (at room temperature for εCL and δVL, but at 40 °C for PDL) followed by vacuum distillation and stored inside the glovebox. For the chemical recycling experiment, PET was introduced in the form of a 20 mL scintillation vial (Fischer Scientific), the PP sample was cut from a centrifuge tube (Sigma Aldrich), and PE was obtained from packaging film used for the scintillation vials. For mechanical testing, films of LDPE (melt flow index = 5 g (10 min)^-1^) and HDPE (melt flow index = 8 g (10 min)^-1^) were prepared using a hot press at 1 MPa and about 10 °C above the melting temperature for 7 minutes.

## Instrumentation

*Size exclusion chromatography (SEC)*

SEC elugrams displaying refractive index *versus* retention time or volume, were obtained on a Malvern GPCMAX instrument containing a PLgel 5 μm guard column (7.5 x 50 mm) and two PLgel 5 μm MIXED-D (300 x 7.5 mm) columns which are kept at a temperature of 35 °C. HPLC grade chloroform containing 2% (v/v) toluene was used as eluent. Polystyrene standards with narrow dispersity (162-364,000 g mol^-1^) were used as calibration. Samples were prepared at ~3 mg mL^-1^ material for a 100 μL injection volume. The flow rate was kept at 0.5 mL min^-1^.

### *Nuclear magnetic resonance (NMR)*

All NMR spectra were recorded in CDCl_3_ on a 400 MHz Bruker Avance III HD instrument at room temperature with TopSpin software. Chemical shifts are reported in parts per million (ppm) and referenced to the residual solvent signal (CHCl_3_: ^1^H, δ = 7.26 ppm, ^13^C, δ = 77.16 ppm).

### *Tensile testing*

Polymer films were prepared by solvent casting from CHCl_3_, and samples were cut from the film with dimensions: w x h x t = 1 cm x 5 cm x 0.3 mm using a scalpel. The samples were mechanically characterized using an Instron 4944 tensile tester with a 500 N load cell. A gauge length of 3 cm with an elongation rate of 6 cm min^-1^. At least five specimens per polymer sample were tested.

### *Differential scanning calorimetry (DSC)*

Thermal properties were recorded using a Mettler Toledo DSC 1 instrument. Aluminium crucibles were loaded with 4-8 mg of each sample and analysed versus an empty crucible. The experiment consisted of two heating scans and two cooling scans starting at -30 °C and proceeding to 200 °C at a heating rate of 10 °C min^-1^ in nitrogen atmosphere.

### *Thermal gravimetric analysis (TGA)*

Thermal degradation of the polymers in a nitrogen atmosphere was analysed on a Mettler Toledo TGA/DSC 1 instrument. For each run, 6-10 mg of sample was loaded in alumina crucible and heated from 35-700°C with a heating rate of 10 °C min^-1^ and a nitrogen flow rate of 20 mL min^-1^.

### *Electron spray ionization – mass spectrometry (ESI-MS)*

The exact mass of the isolated rings was recorded on a Bruker amaZon speed RF 0212G003 2013 spherical ion trap operated using TrapControl 8.0.25 (Bruker Daltonik) and analysed with DataAnalysis 4.0 (Bruker, Bremen, Germany). Samples were prepared by dissolution in HPLC grade MeCN at room temperature, after which they were filtered through a 0.45 µm PTFE filter prior to injection for analysis.

## Methods

### Equilibrium studies

A typical experiment to establish the equilibrium ring concentrations was performed as follows. Inside a nitrogen filled glovebox an oven dried glass vial was charged with δVL (0.2 g, 2 mmol, 100 eq), then toluene (1.33 mL) and *^t^*BuOK 1 M in THF solution (16 mg, 0.02 mmol, 1 eq) were added. The vial was then equipped with a septum seal and transferred outside the glovebox onto a preheated sand bath at 120 °C. After 3 hours, a sample was withdrawn through the septum and dissolved in chloroform containing small amounts of acetic acid to quench the reaction. The sample is then used directly for SEC preparation. The reaction temperature was then lowered by 15 °C and left for at least 3 hours before another sample was withdrawn and evaluated. This process was continued until a temperature of 0 °C, where the final sample is drawn.

### Polymer synthesis

All polymers used in this study were synthesized inside a glovebox as follows. The desired monomer is dissolved in toluene, usually to achieve a concentration of 2 M, and then treated with a desired amount of the *^t^*BuOLi initiator and heated to 90 °C until completion. The crude is then dissolved in CHCl_3_ and the polymer precipitated in MeOH. The polymer is collected by filtration and dried *in vacuo*.

For the polymer used in Figure S2, for example, PDL (2 g, 8.32 mmol, 100 equiv.) was dissolved in toluene (4.16 mL) and then treated with *^t^*BuOLi 1 M in THF solution (83 µL, 0.0832 mmol, 1 equiv.). The sample is then heated to 90 °C for 1 hour, after which it is dissolved in CHCl_3_ and the polymer precipitated in MeOH. The white precipitate is filtered and dried *in vacuo* to yield PPDL (1.84 g, 92%).

### Recycling of PεCL from mixed waste

PεCL with a molecular weight of 129 kg mol^-1^ was synthesized as described in the polymer synthesis section. 3 g of this polymer was then mixed in with PP originating from a falcon centrifuge tube, PET from a commercial PET bottle, and PE from a plastic bag in a glass jar. The jar was filled with 60 mL toluene and heated to 50 °C in a sand bath until all PεCL had dissolved (20 minutes), after which it was treated with *^t^*BuOLi 1 M solution in THF (0.132 mL). The solution was left for 2 hours after which it was filtered through a silica pad. The filtrate was collected and concentrated *in vacuo* to yield the rings as a white/see through gel (2.79 g, 93%). The toluene was also collected after evaporation, where 57 mL of solvent were recovered. The obtained rings were then transferred to a glovebox and used directly for repolymerization. Here, the rings (2 g) were diluted with toluene (1 mL), treated with ethylene glycol dissolved at 2 M in THF (22 µL), and AlEt_3_ dissolved at 1 M in hexanes (9 µL). The solution was then heated to 120 °C for 16 hours after which it was cooled to room temperature, dissolved in CHCl_3_ and dropwise added to a beaker of MeOH. The precipitated was collected and dried in vacuo to yield the recycled PεCL as a white solid (quantitative).

### Data analysis

The SEC traces were integrated directly from the refractive index response over the elution volume. First, the entire area occupied by the rings and linear polymer is established (A_tot_). Then the area of the individual rings (A_i_), the total ring area (A_rings_), and the total linear polymer area (A_polymer_) are calculated. The weight fraction is then calculated through:

$$wt._{i}=\frac{A_{i}}{A_{tot}}$$

$$w{t.}_{ring}=\frac{A_{rings}}{A_{tot}}$$

$$w{t.}_{polymer}=\frac{A_{polymer}}{A_{tot}}$$

Where wt. is the weight fraction and i refers to the size of the rings in terms of monomer units (*e.g.* monomer i = 1, dimer i = 2, etc.). Note that [M]_0_ refers to starting monomer concentration (δVL, PDL, or εCL) meaning that i = 1. To calculate the concentration of an individual ring, the size of the rings needs to be considered yielding:

$$\left[ M \right]_{i}=\frac{A_{i}}{A_{tot}}*\frac{\left[ M \right]_{0}}{i}$$

These equilibrium concentrations are used to calculate the thermodynamic parameters through:

$$\frac{\Delta H}{T}-\Delta S=Rln\left[ M \right]_{i}$$

where a trend line is established between 1/T and Rln[M]_i_ to calculate ∆H form the slope and ∆S from the intersect of the line.

## Additional data


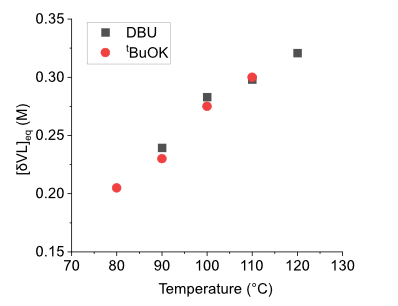


Figure S1. Equilibrium δVL concentration at different temperatures in the presence of different catalysts determined using ^1^H NMR.

*Figure S2. (A) Depolymerization of PPDL (M_n_ = 67.3 kg mol^-1^) over time with ^t^BuOK. (B) M_n_ with time for ^t^BuOK and TBD at 80 °C.*


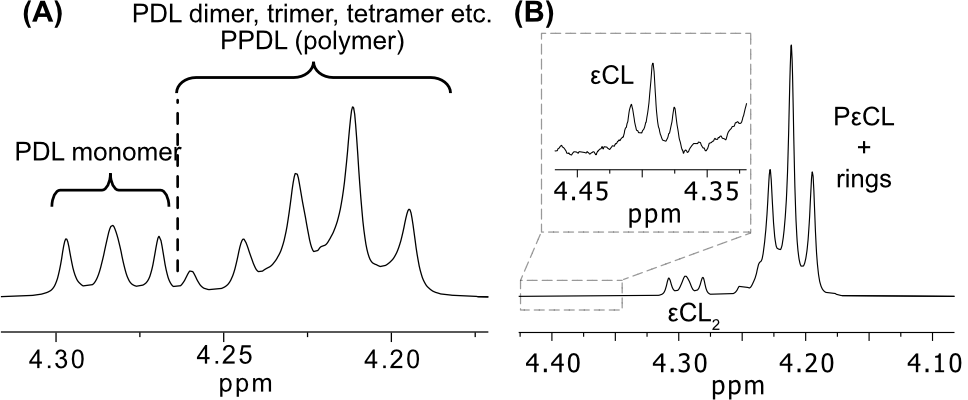


*Figure S3. ^1^H NMR shift of the terminal methylene CH_2_ group during the polymerization initiated with ^t^BuOK of (A) PDL and (B) εCL.*

*Figure S4. Ratio of calculated equilibrium concentration between ^1^H NMR and SEC for δVL, εCL, and PDL monomers in toluene at different temperatures. In each case, monomer concentration was obtained from integration of the monomer signal compared to the signals of monomer+other rings+polymer.*

*
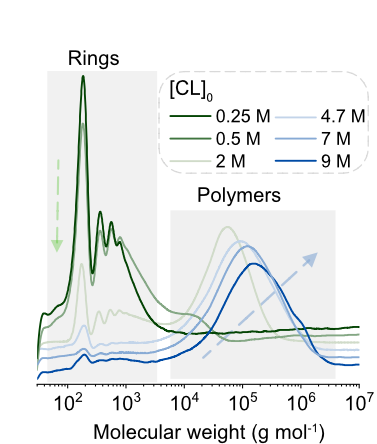
*

Figure S5. SEC elugrams of the obtained RCE during the polymerization of εCL with ^t^BuOK at different starting concentrations.

Figure S6. SEC traces of the equilibrium states of (a) [εCL]_0_=0.25 M, (b) [PDL]_0_=0.25, and (c) [εCL]_0_=2 M at different temperatures.


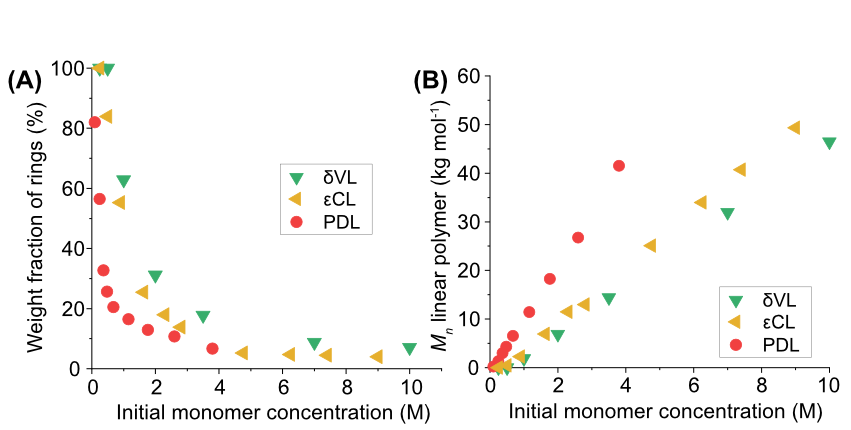


Figure S7. (A) Overlay of ring weight fractions from Figure 3 (B)-(D). (B) M_n_ of polymer chains with increasing monomer starting concentration.

Figure S8. Equilibrium concentration versus inverse temperature plots for determination of thermodynamic parameters for a) δVL b) εCL,, and c) PDL.


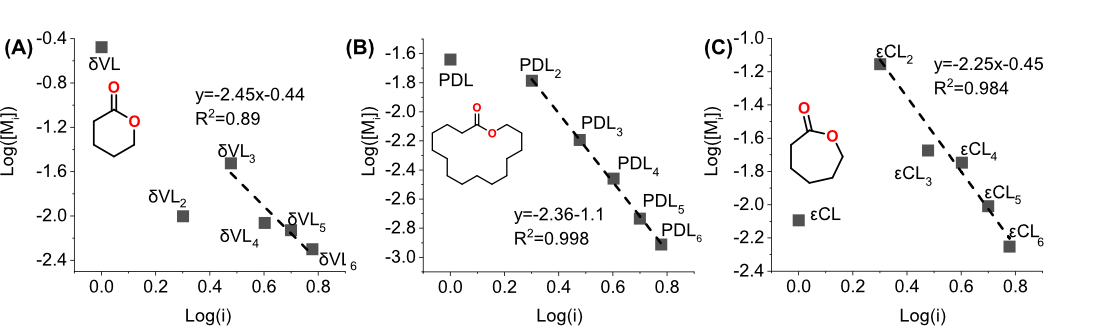


Figure S9. Log-log plots of equilibrium concentration versus ring size (i) at 100 °C for (A) δVL, (B) PDL, and (C) εCL.


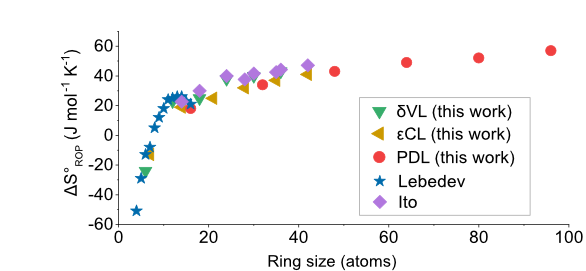


Figure S10. The entropy of ROP for the rings of various sizes compared to literature values for unsubstituted aliphatic lactones. Literature data can be found in Ref. 1 and 2.


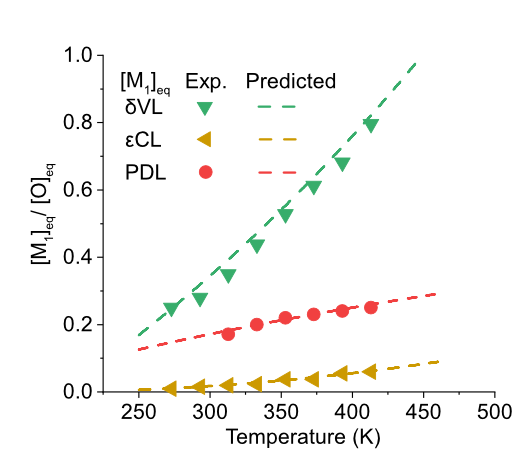


Figure S11. Fraction of monomer out of all rings at equilibrium at different temperatures. Predicted lines were derived from thermodynamics (∆H and ∆S).


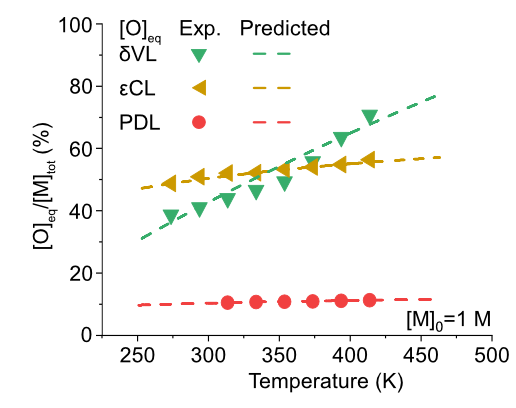


Figure S12. Concentration of monomer present in rings ([O]) at equilibrium for different temperatures. Predicted lines were derived from thermodynamics (∆H and ∆S).


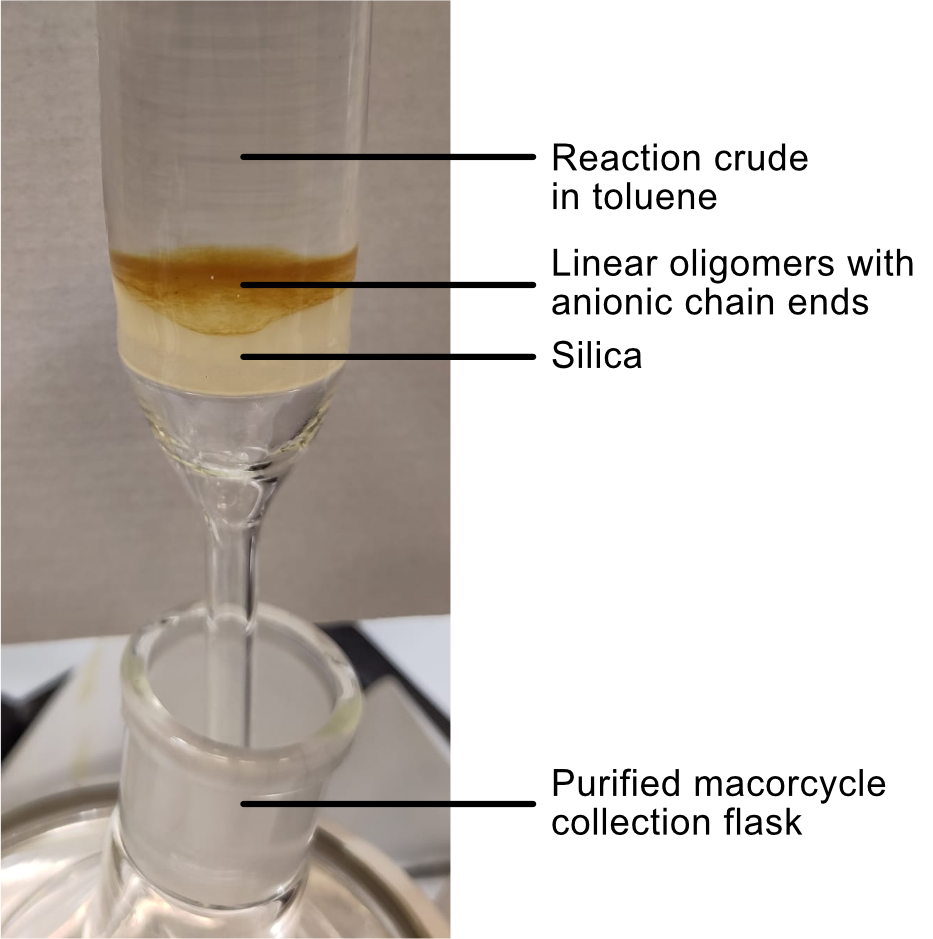


Figure S13. Filtration of reaction crude containing rings and short linear oligomers to remove linear oligomers.

Figure S14. ESI-MS mass spectra of isolated (A) δVL rings, (B) PεCL rings, and (C) PDL rings.

Figure S15. ESI-MS mass spectra of isolated rings obtained from PPDL-PδVL co-depolymerization.

Table S1. M/z values found in figure S13 with proposed composition and counter ion.

| m/z found (g mol^-1^) | m/z theoretical (g mol^-1^) | Composition | Ion |
| --- | --- | --- | --- |
| 274.2 | 274.150 | PDL_2_ | Li_2_-(H_2_O)_3_ |
| 288.05 | 288.200 | PDL | MeCN-Li |
| 318.25 | 318.218 | PDLVL_3_ | K_2_-H_2_O |
| 323.12 | 323.150 | VL_3_ | Na |
| 339.1 | 339.188 | VL_3_ | K |
| 363.24 | 363.260 | PDLVL | Na |
| 379.21 | 379.298 | PDLVL | K |
| 439.18 | 439.238 | VL_4_ | K |
| 463.33 | 463.310 | PDLVL_2_ | Na |
| 479.32 | 479.348 | PDLVL_2_ | K |
| 503.45 | 503.420 | PDL_2_ | Na |
| 579.37 | 579.398 | PDLVL_3_ | K |
| 619.48 | 619.508 | PDL_2_VL_2_ | K |
| 719.54 | 719.558 | PDL_2_VL_3_ | K |
| 819.59 | 819.608 | PDL_2_VL_4_ | K |
| 859.67 | 859.718 | PDL_3_VL | K |
| 919.6 | 919.658 | PDL_2_VL_4_ | K |
| 959.75 | 959.768 | PDL_3_VL_2_ | K |
| 1059.82 | 1059.818 | PDL_3_VL_3_ | K |
| 1159.87 | 1159.868 | PDL_3_VL_4_ | K |
| 1259.91 | 1259.918 | PDL_3_VL_5_ | K |


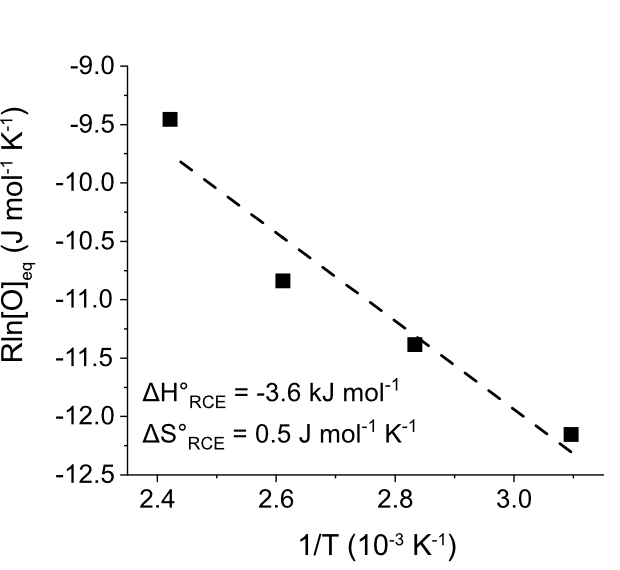


Figure S16. van ‘t Hoff plot for the determination of the enthalpy and entropy of the RCE during co-depolymerization of PδVL and PPDL.

Figure S17. Van ‘t Hoff thermodynamic plot for the determination of δVL ROP parameters during homo polymerization and co-polymerization with PDL systems. Reactions were performed in toluene with 1 mol% ^t^BuOK as initiator.

Table S2. All possible rings that can be formed up to 80 atoms during RCE for PDL-co-δVL, δVL homo, and PDL homo (de)polymerization.

| PDL-co-δVL RCE | | δVL homo RCE | | PDL homo RCE | |
| --- | --- | --- | --- | --- | --- |
| Composition | Atoms in ring | Composition | Atoms in ring | Composition | Atoms in ring |
| VL | 6 | VL | 6 | PDL | 16 |
| VL2 | 12 | VL2 | 12 | PDL2 | 32 |
| PDL | 16 | VL3 | 18 | PDL3 | 48 |
| VL3 | 18 | VL4 | 24 | PDL4 | 64 |
| PDLVL | 22 | VL5 | 30 | PDL5 | 80 |
| VL4 | 24 | VL6 | 36 |  |  |
| PDLVL2 | 28 | VL7 | 42 |  |  |
| VL5 | 30 | VL8 | 48 |  |  |
| PDL2 | 32 | VL9 | 54 |  |  |
| PDLVL3 | 34 | VL10 | 60 |  |  |
| VL6 | 36 | VL11 | 66 |  |  |
| PDL2VL | 38 | VL12 | 72 |  |  |
| PDLVL4 | 40 | VL13 | 78 |  |  |
| VL7 | 42 |  |  |  |  |
| PDL2VL2 | 44 |  |  |  |  |
| PDLVL5 | 46 |  |  |  |  |
| VL8 | 48 |  |  |  |  |
| PDL3 | 48 |  |  |  |  |
| PDL2VL3 | 50 |  |  |  |  |
| PDLVL6 | 52 |  |  |  |  |
| PDL3VL | 54 |  |  |  |  |
| VL9 | 54 |  |  |  |  |
| PDL2VL4 | 56 |  |  |  |  |
| PDLVL7 | 58 |  |  |  |  |
| PDL3VL2 | 60 |  |  |  |  |
| VL10 | 60 |  |  |  |  |
| PDL2VL5 | 62 |  |  |  |  |
| PDLVL8 | 64 |  |  |  |  |
| PDL4 | 64 |  |  |  |  |
| PDL3VL3 | 66 |  |  |  |  |
| VL11 | 66 |  |  |  |  |
| PDL2VL6 | 68 |  |  |  |  |
| PDLVL9 | 70 |  |  |  |  |
| PDL4VL1 | 70 |  |  |  |  |
| PDL3VL4 | 72 |  |  |  |  |
| VL12 | 72 |  |  |  |  |
| PDL2VL7 | 74 |  |  |  |  |
| PDLVL10 | 76 |  |  |  |  |
| PDL4VL2 | 76 |  |  |  |  |
| PDL3VL5 | 78 |  |  |  |  |
| VL13 | 78 |  |  |  |  |
| PDL2VL7 | 80 |  |  |  |  |
| PDL5 | 80 |  |  |  |  |


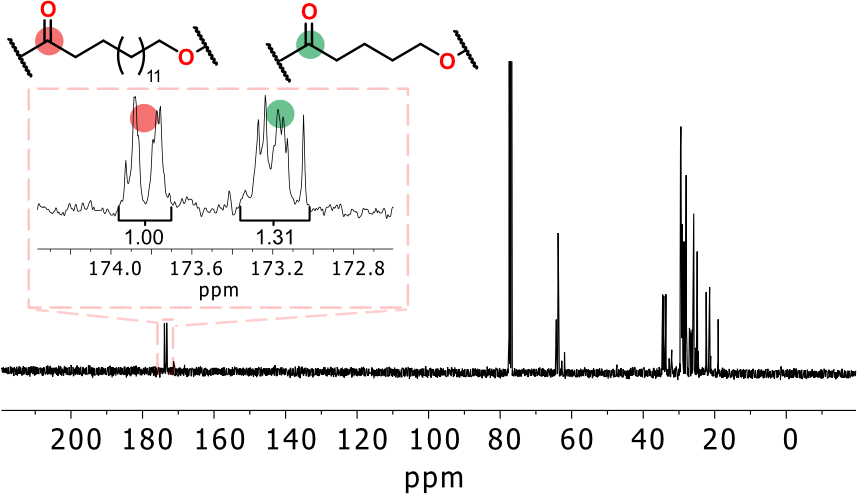


Figure S18. ^13^C NMR spectra (101 MHz, CDCl_3_) of isolated δVL-PDL rings during simultaneous depolymerization of a mixture of PδVL and PPDL in the presence of ^t^BuOK in toluene.


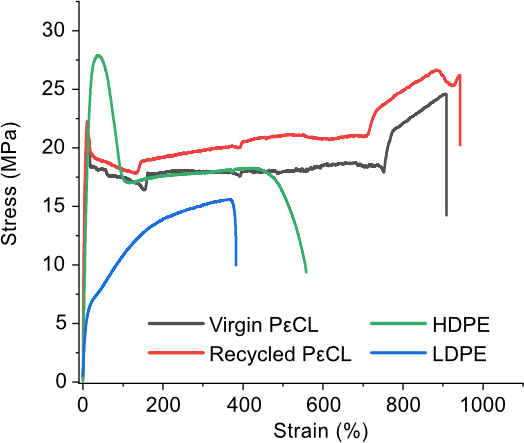


Figure S19. Stress-strain curves of virgin PεCL, recycled PεCL, HDPE (MFI = 8), and LDPE (MFI = 5) from tensile testing (6 mm/min, 298 K).

Figure S20.Raw tensile curves (6 mm/min, 298 K) for (A) HDPE, (B) LDPE, (C) recycled PεCL, and (D) virgin PεCL.


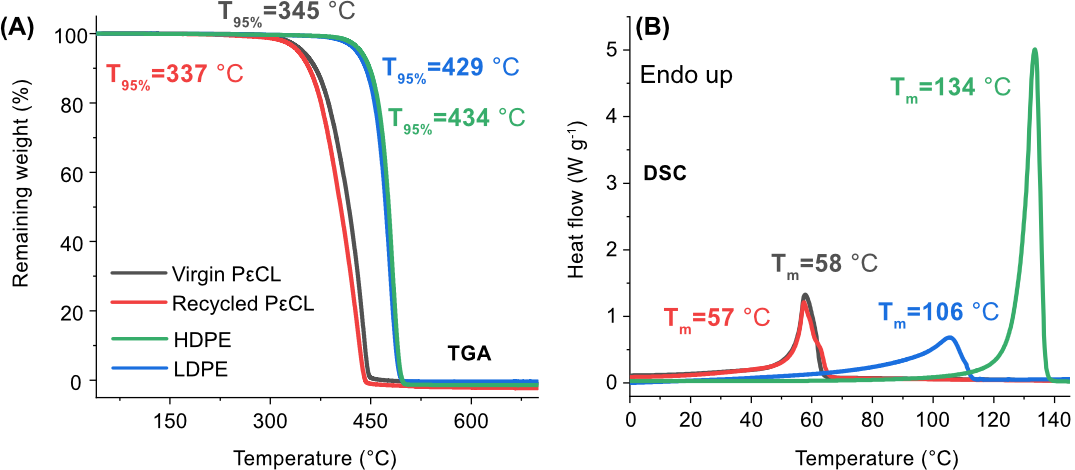


Figure S21. Thermal analysis of PεCL, recycled PεCL, LDPE, and HDPE using (A) TGA (N_2_, 10 K min^-1^) and (B) DSC (N_2_, 10 K min^-1^).

References

(1) Lebedev, B. V. Thermodynamics of Polylactones. *Russ. Chem. Rev.* **1996**, *65* (12), 1063. https://doi.org/10.1070/RC1996v065n12ABEH000265.

(2) Ito, K.; Hashizuka, Y.; Yamashita, Y. Equilibrium Cyclic Oligomer Formation in the Anionic Polymerization of Iε-Caprolactone. *Macromolecules* **1977**, *10* (4), 821–824. https://doi.org/10.1021/ma60058a019.
